# X-ray lasers for structure and dynamics in biology

**DOI:** 10.1107/S2052252518005365

**Published:** 2018-04-12

**Authors:** John C. H. Spence

**Affiliations:** a Physics, Arizona State University, Tempe 85287-1504, USA

**Keywords:** X-ray lasers, XFELs, protein dynamics, structural biology

## Abstract

Recent advances in the application of X-ray lasers to structural biology are providing time-resolved high-resolution imaging of many processes, from enzyme kinetics to the riboswitch in action, drug action, and light-sensitive proteins.

It is now almost a decade since the first hard X-ray laser started operation at the Department of Energy’s SLAC laboratory in the United States, soon followed by its first use in biology. In that time we have seen some dramatic advances in structural biology as a result, as reviewed, for example, in Schlichting (2015 ▸), Bostedt *et al.* (2016 ▸) and Spence (2017 ▸). We have also seen the start of operation of four additional machines in Japan (SACLA), Switzerland (SwissFEL), South Korea (PAL) and Germany (EuXFEL). A compact XFEL (CXFEL) is under construction on the campus of Arizona State University. The machines produce pulses of hard X-rays, each containing up to 10^12^ photons per pulse in a matter of femoseconds, so brief as to out-run most radiation damage effects. Diffraction patterns are read out at kilohertz rates, one for each pulse. The sample is destroyed following termination of the incident pulse and the elastic scattering it generates. This has allowed structures to be determined from snapshots taken at room temperature, under near-physiological conditions, opening up the field of protein dynamics and the direct imaging of intermediate states during protein dynamics. For crystals, the method of serial crystallography (SC) is used, in which a stream of hydrated microcrystals flows across the beam. Serial crystallography has also been developed at synchrotrons, using both viscous delivery media and liquid jets, where the source brightness and detector speed may be sufficient to freeze microcrystal rotation during an exposure as they flow across the beam. The crystal orientation, data merging and structure factors, are all obtained using the new algorithms developed for serial crystallography. Phasing is generally undertaken using molecular replacement or SAD; however, novel methods based on the unique capabilities of the XFEL are also under development.

Advances have been achieved in several areas, from a program of structure-based drug design based on XFEL analysis, to time-resolved imaging of light-sensitive proteins and, most recently, the first atomic resolution time-resolved images of chemical dynamics, opening the way to direct imaging of processes such as enzyme kinetics. In parallel with these studies based on micron-sized protein crystals, work on single-particle (SP) methods (with one non-crystalline bioparticle, such as a virus, per shot) has progressed steadily, most recently producing images of a virus capsid in the process of ejecting its genome, and mapping out the energy landscape for this process. An exciting development in this area has been the recent proposal to use the Hanbury–Brown and Twiss effect to image inner-shell fluorescence from heavy atoms in proteins. It can be shown that fluorescence from different atoms is coherent for incident pulses briefer than the lifetime of the excitation, and this idea is currently being tested at LCLS beamtimes. Fast solution scattering (FSS) has seen similar progress using pump-probe methods, while angular correlation techniques have finally proven useful for single particles, based on the early ideas of Z. Kam.

In the field of drug design, at least ten GPCR structures have now been solved, forming the basis for a program of structure-based drug design based on XFEL results and related methods. In total over a hundred structures have been deposited in the PDB based on XFEL results, including rhodopsin bound to arrestin, and cytochrome *c* oxidase, while the structures of lysozyme, glucose isomerase, thaumatin and fatty acid-binding protein type 3 have also been reported at a resolution of better than 2 Å, among many others, including a study of nitrite reductase. An important discovery has been that diffuse scattering due to static molecular displacements can be used both to extend resolution and assist phasing.

Light-sensitive proteins allow use of a fast optical trigger, providing high time resolution, with atomic resolution imaging (in crystals), not possible by other methods. The ‘movies’ which result from these pump-probe studies show the amount of the various molecular species present in the crystal as the reaction proceeds, based on analysis of Bragg intensities, and hence on ensemble averages. These may be fitted to the appropriate rate equations to provide rate coefficients and barrier heights. In one remarkable study of Purple Yellow Protein, akin to the first event in human vision, the *cis*–*trans* isomerization reaction (a conical intersection) which occurs when a protein absorbs a photon has been imaged at seven time intervals with 150 fs time resolution. Work on photosynthesis continues by similar methods in this highly competitive field, aimed at time-resolved imaging of the atomic mechanism for water splitting and sugar synthesis. X-ray emission spectroscopy is commonly used in synchrony with the collection of snapshot diffraction patterns. Time-resolved serial crystallography using an optical trigger has also been undertaken using viscous delivery media such as the lipid cubic phase, which has become a popular sample delivery method because it also provides the growth medium for the microcrystals, including membrane proteins.

A new development has been the use of mixing jets to allow time-resolved imaging of chemical reactions triggered by, for example, a substrate diffusing into an enzyme in crystalline form. This ‘mix-and-inject’ method, where flowing microcrystals mix with substrate prior to injection across the beam, takes advantage of the small size of the microcrystals and resulting rapid diffusion of small molecules throughout them. One then has both the advantages of atomic resolution which Bragg diffraction provides, while keeping the diffusion time in the crystal (longer than in solution) below the reaction time, so that it is not rate-limiting. By changing the reaction time prior to the snapshot for each frame, a time series of Bragg diffraction patterns may be recorded for many crystal orientations, producing a three-dimensional ‘movie’ of the chemical reaction. Examples include the binding of the antibiotic drug ceftriaxone (used against tuberculosis) with the enzyme β-lactamase, and the observation of intermediate structures of the RNA riboswitch during regulation of gene expression. Many more studies of this type are in progress, which are bound to produce striking new results in the near future relevant to structural enzymology and the use of intermediates as drug targets, among others.

Advances in this field are limited currently by the development of new microcrystal growth methods, new sample delivery methods, detector developments, and the need for improved algorithms which can separate the X-ray scattering contributions of several species in a crystal whose molecules are undergoing a chemical reaction. The current literature abounds with new ideas in all these areas, from conveyor-belt delivery systems to machine-learning algorithms and detectors capable of increasing dynamic range at each pixel during data collection. For the imaging of protein dynamics especially, where femtosecond pulses can minimize the electronic reduction of species by the beam during a reaction, yet provide high-resolution ‘movies’ over time intervals from femtoseconds to milliseconds, we expect (using all the modalities discussed above) dramatic advances in the near future. We welcome authors to share these exciting developments in **IUCrJ**, which has reported many of the developments and results during the last 4 years.
